# Editorial: Investigating the gliomas/white matter interplay and its implications for multidisciplinary treatment: State of art and future perspectives

**DOI:** 10.3389/fnins.2022.1100972

**Published:** 2022-12-08

**Authors:** Francesco Latini, Asgeir Jakola, Roberta Rudà

**Affiliations:** ^1^Section of Neurosurgery, Department of Medical Sciences, Uppsala University, Uppsala, Sweden; ^2^Department of Neurosurgery, Sahlgrenska University Hospital, Gothenburg, Sweden; ^3^Division of Neuro-Oncology, Department of Neuroscience “Rita Levi Montalcini”, University of Turin, Turin, Italy

**Keywords:** brain connectome networks, diffuse gliomas, white matter, brain mapping, neuroplasticity, advanced neuroimaging, individualized oncological treatment

Important conceptual and methodological advances have been achieved in the last decades with a significantly better understanding of the anatomic and functional connectivity of the brain (Elam et al., 2021). However, how the structural and functional organization of the host brain adapts/reacts to a brain tumor is still poorly understood (Duffau, 2021).

Gliomas are heterogeneous and challenging tumors, they tend to preferentially infiltrate “secondary” functional areas (immediately near the so-called primary eloquent regions), or the so-called “minimal common brain” (Duffau and Capelle, 2004; Ius et al., 2011; Latini et al., 2020). It is clear that the tumor tends to interfere with normal brain function by disrupting the functional connectivity of brain networks within peritumoral and distant brain areas, thereby inducing neuropsychological impairment or seizure activity (Szalisznyo et al., 2013; Smits et al., 2015; Smits and Jakola, 2019; Aabedi et al., 2021; Latini et al., 2021a). The mechanisms of cortical-subcortical plasticity at the individual level are not fully understood with important anatomical-functional variability (Herbet et al., 2016; Sarubbo et al., 2020). The subcortical white matter bundles often represent the main limit for a surgical resection and, at the same time, they represent the way of least resistance that glioma cells use to disseminate (Engwer et al., 2015; Alfonso et al., 2017; Latini et al., 2021b). A better comprehension of the white matter interaction with gliomas became pivotal in several fields of neurosurgical oncology to improve diagnosis, treatment, and outcome.

This Research Topic includes five articles that investigated some of the major themes and developments in the white matter/gliomas interplay: (1) Radiological advances in neuroimaging of white matter; (2) Effect of radiotherapy on patients performance in a high eloquent area; (3) Clinical/surgical implication of diffuse gliomas in eloquent areas; (4) White matter plasticity.

Two articles in this issue focused on neuroimaging of white matter in patients with gliomas. Brabec et al. in an original study investigated tumor-related hyperintensities and white matter signals with both linear and spherical tensor encoding (LTE and STE). Their goal was to investigate whether STE could be used to suppress white matter signals and enhance the conspicuity of glioma hyperintensities unrelated to white matter. The results of this study suggest that STE-DWI may be relevant in clinical settings for diagnosis or radiotherapy planning, where the presence of a pathology-related hyperintensity may improve detection or delineation. STE-DWI could also be useful to visualize tumor-related hyperintensities within white matter tracts which may be not visible with LET-DWI, contributing to the differential diagnosis between shifted/dislocated and infiltrated white matter fibers. The authors found that the highest signal differences between LTE-DWI and STE-DWI were observed predominately in major white matter tracts suggesting that the inclusion of STE-DWI in as a complementary diffusion sequence would be able to better show lesions “hidden” under white matter in these tracts.

Advances in neuroimaging of white matter in gliomas were also described by Friedrich et al., who investigated the impact of different types of damage on the fiber architecture of the affected white matter in patients treated for gliomas. The authors analyzed patients during the post-treatment follow-up with morphological MR, O-(2-[18F]fluoroethyl)-L-tyrosine (FET) PET images, and Constrained spherical deconvolution (CSD) algorithm to detect fiber density. They also compared the results with the patients' global performance status. They found that the structural and metabolic imaging changes after multimodal therapy in glioma patients were associated with a significant reduction in local white matter fiber density. The total fiber loss in contrast-enhancing lesions and T2/FLAIR hyperintense regions was associated with a significant risk of lowered performance status, while the total fiber loss caused by resection and regions with increased FET uptake did not impact general performance. This study suggests that apart from resection cavities, reduction in local fiber density is greatest in contrast-enhancing recurrent tumors, but total fiber loss induced by edema or gliosis or damage related to radiotherapy has an equally detrimental effect on the patients' performance status due to the large-scale white matter networks affected.

The impact of oncological treatment in gliomas on performance status is even more important in cases where the surgery is not possible for instance in diffuse intrinsic pontine glioma (DIPG). This aspect was considered by Chavaz et al., which investigated the spectrum of neurological triad improvement (cranial nerve deficits, ataxia, and long tract signs) in patients with DIPG treated by re-irradiation (re-RT). Patients at first progression were assessed by clinical benefits on neurological triad and were categorized as “responding” or “non-responding” to re-RT. The authors conclude that a median re-irradiation dose of 20 Gy may provide a neurological benefit in two-thirds of patients with an improvement of at least one symptom of the triad, but the survival from the start of re-RT to death was not different between responding and non-responding DIPG patients.

On the other hand, for supratentorial gliomas, the possibility to achieve a complete surgical resection remains one of the most important prognostic factors. A total or supra-total resection in tumors harboring language areas/networks still represents one of the major challenges in neurosurgical practice. Aabedi et al., summarized in a review paper the actual knowledge regarding the clinical implications of gliomas involving white matter language tracts. In this article, the authors discussed the advantages and limitations of methods commonly used to identify white matter tracts during surgery proposing a multimodal mapping paradigm to optimize postoperative language outcomes. This review also provided a discussion on local and long-range adaptations that take place as the language network undergoes remodeling after tumor growth and surgical resection. Some of the probable cellular mechanisms underlying this plasticity were also discussed in this article. Their contribution gives an overview of emerging developments in targeting the glioma-neuronal network interface to achieve better disease control and promote recovery after injury.

One of the most intriguing theories to promote recovery after injury is linked to the possibility to increase white matter plasticity, which has been considered impossible or very low in the past times. In his review article Duffau, provides details regarding the role of myelin plasticity in gliomas. The goal of this review article was to describe the implications of constant interactions between WM tracts and gliomas. The author discusses literature results on the interaction between white matter and gliomas through the myelin status, also concerning the origins of this tumor, the patterns of dissemination of glioma cells, and the functional consequences of glioma infiltration on personalized management. In addition, this article suggests new directions for a more individualized treatment plan and new research targets in patients with gliomas.

This Research Topic provides new insights into neuroimaging techniques, available treatment options, and future directions for individualized treatment in patients with gliomas. The necessity to tailor both surgical resection and irradiation planning based on the patient-specific anatomical/functional organization but also to the infiltration pattern along white matter fibers seems already necessary to improve the onco-functional balance at the individual level (Duffau, 2017; Mandonnet and Duffau, 2018). New neuroimaging techniques have the potential to assist in diagnosis, and predict the infiltration pattern. Preoperative and intraoperative mapping techniques of the individual brain connectome can optimize surgical and post-surgical treatment. The possibility to identify new therapeutic targets able to inhibit glioma invasion and promote myelin plasticity are the new challenges to improve both the functional and oncological outcome of these patients.

Further studies are needed in all the fields of neuro-oncology to achieve better results for these patients. Only a multimodal, multidisciplinary and open-minded approach to this disease would be able to decode and change the interplay between brain white matter and gliomas.

## Author contributions

All authors listed have made a substantial, direct, and intellectual contribution to the work and approved it for publication.

## References

[B1] Aabedi A. A. Lipkin B. Kaur J. Kakaizada S. Valdivia C. Reihl S.. (2021). Functional alterations in cortical processing of speech in glioma-infiltrated cortex. Proc. Natl. Acad. Sci. U. S. A. 118, e2108959118. 10.1073/pnas.2108959118 34753819 PMC8609626

[B2] Alfonso J. C. L. Talkenberger K. Seifert M. Klink B. Hawkins-Daarud A. Swanson K. R.. (2017). The biology and mathematical modelling of glioma invasion: a review. J. R. Soc Interface 14, 20170490. 10.1098/rsif.2017.0490 29118112 PMC5721156

[B3] Duffau H. (2017). A two-level model of interindividual anatomo-functional variability of the brain and its implications for neurosurgery. Cortex 86, 303–313. 10.1016/j.cortex.2015.12.009 26920729

[B4] Duffau H. (2021). Dynamic interplay between lower-grade glioma instability and brain metaplasticity: proposal of an original model to guide the therapeutic strategy. Cancers 13, 4759. 10.3390/cancers13194759 34638248 PMC8507523

[B5] Duffau H. Capelle L. (2004). Preferential brain locations of low-grade gliomas. Cancer 100, 2622–2626. 10.1002/cncr.20297 15197805

[B6] Elam J. S. Glasser M. F. Harms M. P. Sotiropoulos S. N. Andersson J. L. R. Burgess G. C.. (2021). The human connectome project: a retrospective. Neuroimage 244, 118543. 10.1016/j.neuroimage.2021.118543 34508893 PMC9387634

[B7] Engwer C. Hillen T. Knappitsch M. Surulescu C. (2015). Glioma follow white matter tracts: a multiscale DTI-based model. J. Math. Biol. 71, 551–582. 10.1007/s00285-014-0822-7 25212910

[B8] Herbet G. Maheu M. Costi E. Lafargue G. Duffau H. (2016). Mapping neuroplastic potential in brain-damaged patients. Brain 139, 829–844. 10.1093/brain/awv394 26912646

[B9] Ius T. Angelini E. Thiebaut de Schotten M. Mandonnet E. Duffau H. (2011). Evidence for potentials and limitations of brain plasticity using an atlas of functional resectability of WHO grade II gliomas: towards a “minimal common brain.” Neuroimage 56, 992–1000. 10.1016/j.neuroimage.2011.03.022 21414413

[B10] Latini F. Axelson H. Fahlström M. Jemstedt M. Alberius Munkhammar Å. Zetterling M.. (2021a). Role of preoperative assessment in predicting tumor-induced plasticity in patients with diffuse gliomas. JCM 10, 1108. 10.3390/jcm10051108 33799925 PMC7961995

[B11] Latini F. Fahlström M. Behánová A. Sintorn I.-M. Hodik M. Staxäng K.. (2021b). The link between gliomas infiltration and white matter architecture investigated with electron microscopy and diffusion tensor imaging. Neuroimage Clin. 31, 102735. 10.1016/j.nicl.2021.102735 34247117 PMC8274339

[B12] Latini F. Fahlström M. Hesselager G. Zetterling M. Ryttlefors M. (2020). Differences in the preferential location and invasiveness of diffuse low-grade gliomas and their impact on outcome. Cancer Med. 9, 5446–5458. 10.1002/cam4.3216 32537906 PMC7402839

[B13] Mandonnet E. Duffau H. (2018). An attempt to conceptualize the individual onco-functional balance: why a standardized treatment is an illusion for diffuse low-grade glioma patients. Crit. Rev. Oncol. Hematol. 122, 83–91. 10.1016/j.critrevonc.2017.12.008 29458793

[B14] Sarubbo S. Tate M. De Benedictis A. Merler S. Moritz-Gasser S. Herbet G.. (2020). Mapping critical cortical hubs and white matter pathways by direct electrical stimulation: an original functional atlas of the human brain. Neuroimage 205, 116237. 10.1016/j.neuroimage.2019.116237 31626897 PMC7217287

[B15] Smits A. Jakola A. S. (2019). Clinical presentation, natural history, and prognosis of diffuse low-grade gliomas. Neurosurg. Clin. N. Am. 30, 35–42. 10.1016/j.nec.2018.08.002 30470403

[B16] Smits A. Zetterling M. Lundin M. Melin B. Fahlström M. Grabowska A.. (2015). Neurological impairment linked with cortico-subcortical infiltration of diffuse low-grade gliomas at initial diagnosis supports early brain plasticity. Front. Neurol. 6, 137. 10.3389/fneur.2015.00137 26113841 PMC4462100

[B17] Szalisznyo K. Silverstein D. N. Duffau H. Smits A. (2013). Pathological neural attractor dynamics in slowly growing gliomas supports an optimal time frame for white matter plasticity. PLoS ONE 8, e69798. 10.1371/journal.pone.0069798 23922804 PMC3724895

